# Fish, Seafood, and Fish Products Purchasing Habits in the Spanish Population during COVID-19 Lockdown

**DOI:** 10.3390/ijerph191811624

**Published:** 2022-09-15

**Authors:** Rocío de la Iglesia, Ángela García-González, María Achón, Gregorio Varela-Moreiras, Elena Alonso Aperte

**Affiliations:** 1 Research Group “Alimentación y Nutrición en la Promoción de la Salud (Food and Nutrition in Health Promotion (CEU-NutriFOOD))”, Departamento de Ciencias Farmacéuticas y de la Salud, Facultad de Farmacia, Universidad San Pablo-CEU, CEU Universities, Urbanización Montepríncipe, 28660 Boadilla del Monte, Spain; 2 Research Group “Nutrición para la Vida (Nutrition for Life)”, Departamento de Ciencias Farmacéuticas y de la Salud, Facultad de Farmacia, Universidad San Pablo-CEU, CEU Universities, Urbanización Montepríncipe, 28660 Boadilla del Monte, Spain

**Keywords:** SARS-CoV-2, fishery, shellfish, e-commerce, elderly

## Abstract

The Mediterranean diet is a healthy dietary pattern in which fish consumption is an important key element. In Spain, fish intake is the second highest in Europe. Dietary guidelines recommend an intake of 1–3 portions a week of fish. However, Spanish fish sales have been decreasing since 2008. The unexpected pandemic spread of the SARS-CoV-2 in 2020 led the Spanish Government to take restrictive measures that had an impact on people’s behavior, including food purchases and consumption. The aim of the study was to analyze purchase data of fish, seafood, and fish products during the lockdown in Spain, using data from loyalty card holders (>5,000,000 participants) from a hypermarket chain in Spain. The results show a 45% increase in the purchase of all types of fish, seafood, and fish products, with the highest increment observed in the retiree (+65 years) as compared to the younger populations. Moreover, the retiree, in spite of the digital divide, were also the ones that most increased online shopping. These data should be considered since events like COVID-19 confinement can have a permanent impact on people’s dietary habits, a possibility that should be monitored in the future.

## 1. Introduction

The Mediterranean diet is a well-known healthy dietary pattern in which fish consumption is an important element [1]. In this regard, Spain is the second country in the European Union with the highest fish consumption [2]. However, a downward trend in fish sales has been observed in Spain since 2008. Specifically, in the period 2013–2019, there was a 15.7% decrease in the volume of purchases of fishery products, according to the 2019 annual report carried out by the Government Household Food Consumption Panel [3]. These data should not go unnoticed. Although the latest dietary recommendations suggest that diets should be based on vegetables and whole grains [4], between one and three portions a week of fish are recommended according to the latest dietary guidelines for the Spanish population [5,6]. Besides being good protein sources, oily fish contain high amounts of the cardioprotective long-chain n-3 eicosapentaenoic and docosahexaenoic fatty acids and are considered the main dietary source of vitamin D in the Spanish population. In addition, small fish species that are eaten whole are also important dietary sources of calcium [7].

The unexpected pandemic spread of the SARS-CoV-2 virus in 2020 causing the COVID-19 disease wreaks havoc worldwide. In Spain, on 14 March 2020, the Government declared a state of alarm for the management of the health crisis situation due to the increasing number of deaths related to the COVID-19 pandemic (Royal Decree 463/2020). Consequently, from 15 March to 21 June 2020, hard lockdown measures were implemented. Spanish citizens were only allowed to leave home to buy food and medicines from supermarkets and pharmacies, visit healthcare units, or go out to work if essential workers. Meanwhile, schools and universities, pubs and restaurants, and all stores except groceries and pharmacies remained closed. This produced a sudden change in the daily routines of the Spanish households, including, almost certainly, changes in shopping behaviors and fishery purchases. 

From our point of view, the analysis of purchases during the months of hard lockdown is of special interest since events like COVID-19 confinement can have a permanent impact on people’s behavior. Although the lockdown measures have now been relaxed, some trends may persist, such as teleworking, continuous day education at schools, or acquired behaviors, such as shopping online and/or new restaurants’ take away/delivery practices.

Different studies have already revealed some of the effects of the COVID-19 lockdown on general eating behaviors in different countries. In this sense, in a recent systematic review about the impact of the pandemic on food intake, eating behaviors, and diet quality, a total of 95 studies were included [8], seven of them were carried out in Spain. Amongst the whole sample, most of the studies (70%) observed a decrease in fish and seafood intake. However, different changes in consumers’ food practices were seen within and between countries [9]. In fact, contrary to many of the countries studied, seafood and fish intake increased in Spain, according to a Spanish study that revealed data on fish consumption [10].

In addition, some studies have found that the impact of COVID-19 quarantine on dietary behaviors may have been different within the same country, depending on the type of population. Thus, nutritional status, age, or socioeconomic situation could be determinant factors in this regard [11,12]. Nevertheless, most of the studies were web-based surveys. This kind of survey presents two main limitations: Recall bias and reliance on self-reported measurements, and there is a tendency to report healthier dietary habits than those actually practiced [13,14]. An alternative source that can provide unbiased data collection for a large number of individuals is hypermarkets’ loyalty cards, which automatically capture the items the customers buy at the store, avoiding impreciseness or over- or underestimation [14].

Another fact that should be considered is that these prior studies mainly revealed the effects of the COVID-19 lockdown on dietary habits. However, the effect on food purchasing behaviors has been less investigated, even though it can have a permanent impact on the country’s economy. In this respect, a study of more than 1900 Chinese adult residents revealed that people preferred in-person to online shopping, although more than half of the studied population did at least one online or delivery order during the lockdown [15]. Moreover, in a large Canadian study, around 60% of the population used online retailing during the year 2020, either to do groceries or to buy from restaurants [16]. However, in an Italian study, only 9% of the population used online delivery during the pandemic [11].

To date, we have not found any published study focused on the possible effect of the COVID lockdown on fish purchasing habits in Spain, using objective data, such as those generated from loyalty cards, nor analyzing the potential sift of in-person to online purchases, which may have influenced purchase elections. Therefore, the aim of the present study was to analyze purchase data for fish, seafood, and fish products, according to the type of retailing (e-commerce or physical store) and different population groups during the months of severe lockdown in Spain, using loyalty card holders’ data from one of the largest hypermarkets chains in Spain and worldwide.

## 2. Materials and Methods

Records on fish purchases were obtained from loyalty card holders from Carrefour Group, the hypermarket with the second largest sales share in Spain [17]. Carrefour is a French-based multinational retail corporation that operates a chain of hypermarkets, grocery stores, and convenience stores. In Spain, Carrefour centers comprise 205 hypermarkets, 1152 supermarkets, 145 gas stations, and travel agencies.

When customers purchase products and present their loyalty card, the purchase is automatically registered by a central system. Specifically, the dataset of the present study consisted of units (number of items) of fish products purchased by loyalty card holders both at Spanish physical stores and e-commerce from March to June 2019 and the same period in 2020. Food products were then categorized according to the Food Consumption Panel of the Spanish Ministry of Agriculture, Fisheries and Food [18]. Products assessed included fresh and frozen fish, fresh and frozen seafood, cooked seafood, canned fish, and fish-based ready-to-eat meals. Personal data, such as date of birth, sex, and family members, were provided voluntarily by the customers when applying for the loyalty card. Clients were then classified according to the personal information given and considering their purchase choices, as shown in Table 1. All data used were anonymized, and researchers did not have access to personal data.

All statistical analyses were conducted using IBM SPSS 27 software for Windows. Absolute values (total number of purchases) were adjusted by the number of loyalty card holders that made at least one purchase of food products during the periods analyzed (March to June 2019 and March to June 2020). The Student’s t-test was used to compare differences between purchases in 2019 and 2020. Comparisons of the relative change of purchases between different types of fish products, categories of loyalty card holders, and the type of retailing were assessed using one-way ANOVA with the post hoc Tukey’s test. Values of *p* < 0.005 were considered statistically significant.

## 3. Results

Fish, shellfish, and fishery products purchase data from a total of 5,511,285 and 5,145,816 loyalty card holders that made at least one purchase of food products during the years 2019 and 2020, respectively, were collected. On average, 61% were women. The mean age of all clients was 46.8 years.

In 2020, each loyalty card holder that made a purchase at the food section of the hypermarket bought an average of 35.2 units of fish, shellfish, and fishery products during the period of March to June, a significantly higher amount than in 2019, when the average was 24.3 units per loyalty card holder (*p* < 0.001). This represents a 45% increase in purchases.

**Figure 1:**
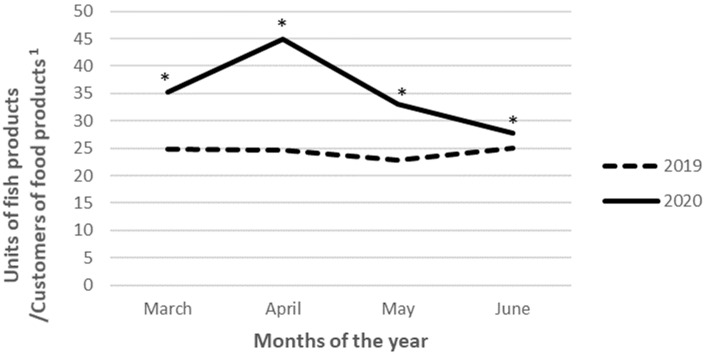
Total purchases of fish, seafood, and fish products by Spanish consumers during the period March to June in 2019 and 2020. * Significant differences (*p* < 0.001) between 2019 and 2020. ^1^ Number of loyalty card holders that made at least one purchase of food products during the periods analyzed.

When analyzing by month, units purchased per loyalty card holder in 2019 did not vary too much along the period (minimum 22.8 units in May vs. maximum 25.0 units in June). However, in 2020 the amount purchased in March was around 10 units higher than in 2019 (24.8 March 2019 vs. 35.2 March 2020, *p* < 0.001). This quantity increased by + 9.8 units in April, making the difference between 2019 and 2020 even greater (24.7 April 2019 vs. 45.0 April 2020, *p* < 0.001). However, purchases in 2020 decreased in May by 11.9 units and continued decreasing by 5.3 units in June. Therefore, although the number of units of fish products purchased per consumer was always significantly higher in 2020 as compared to 2019 (*p* < 0.001), the biggest difference was observed in April, followed by March and May, as shown in Figure 1.

**Figure 2:**
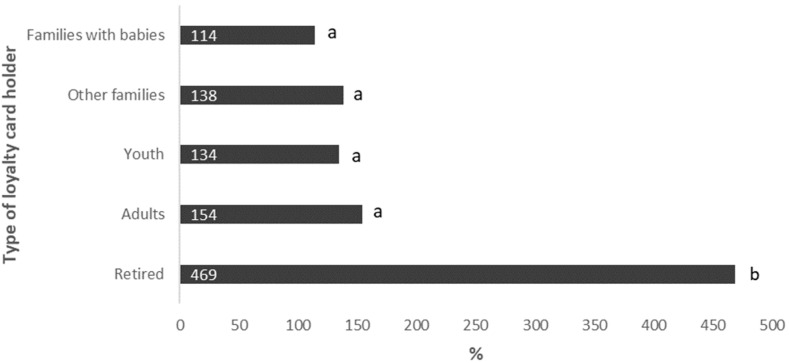
Relative increment in total purchases of fish, seafood, and fish products in Spain, from 2019 to 2020, during the period March to June, according to the type of loyalty card holder in Spain. 11% of loyalty card holders are unclassified. Different letters represent statistically significant differences (*p* < 0.005).

According to the type of loyalty card holder, all categories increased their fish, shellfish, and fishery purchases during the months of the Spanish lockdown as compared with the same period in 2019. However, it is noteworthy that this increment was significantly higher in the “retired” group as compared to the other categories (*p* < 0.001). In fact, while the relative increment of purchases of the categories “families with babies”, “other families”, “youth” and “adults” was between 114 and 154%, this increment reached 469% in the retirees (Figure 2). 

**Figure 3:**
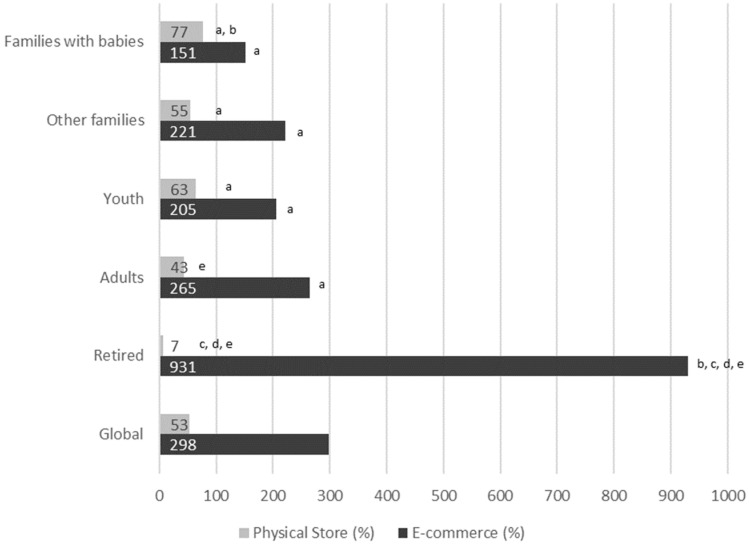
Relative increment in purchases of fish, seafood, and fish products in Spain, from 2019 to 2020, during the period March to June, according to loyalty card holder category and type of retailing. 11% of loyalty card holders are unclassified. In all cases, differences between e-commerce and physical store are statistically significant (*p* < 0.005). Comparative between the type of customers within the same group (E-commerce or Physical store); a, Significant differences compared to elderly; b, Significant differences compared to adults; c, Significant differences compared to youth; d, Significant differences compared to other families; e, Significant differences compared to families with babies.

Regarding the type of retailing, in all cases, the relative increment of purchases was significantly higher in e-commerce as compared with physical stores (*p* < 0.005). Noteworthy, there is a significantly higher increment in online purchases in the retired category of loyalty card holders, as compared with the other four categories (*p* < 0.001), as shown in Figure 3.

**Figure 4:**
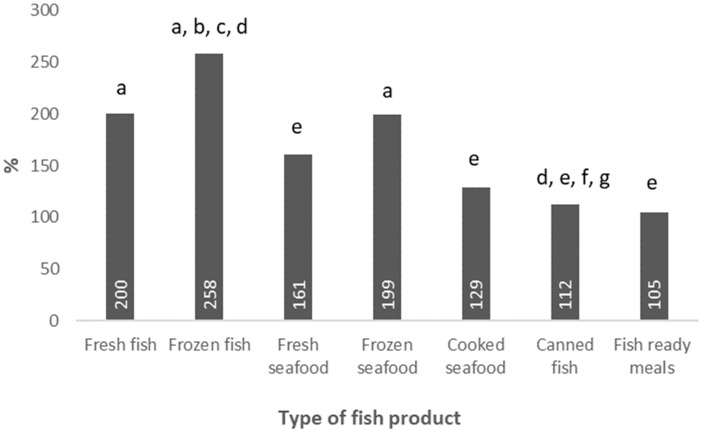
Relative increment in total purchases of fish, seafood, and fish products in Spain from 2019 to 2020, during the period March to June, according to type of fish product. a, Significant differences (*p* < 0.005) compared to canned fish; b, Significant differences (*p* < 0.005) compared to fresh seafood; c, Significant differences (*p* < 0.005) compared to cooked seafood; d, Significant differences (*p* < 0.005) compared to fish based ready-to-eat meals; e, Significant differences (*p* < 0.005) compared to frozen fish; f, Significant differences (*p* < 0.005) compared to fresh fish; g, Significant differences (*p* < 0.005) compared to frozen seafood.

If we focus on the type of product bought, a significant increment of all types of fishery goods purchases was observed, being the highest for “frozen fish” followed by “fresh fish” and “frozen seafood”, while “fish-based ready-to-eat meals” was the category whose sales increased the least, as shown in Figure 4. 

**Figure 5:**
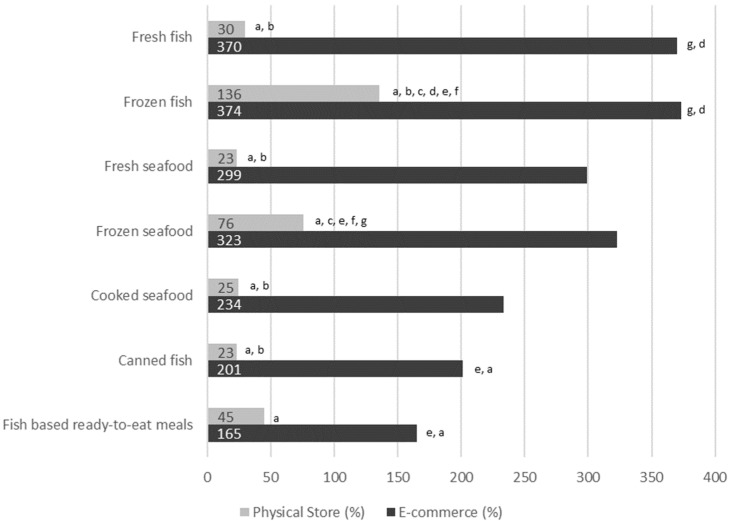
Relative increment in purchases of fish, seafood, and fish products in Spain from 2019 to 2020, during the period March to June, according to fish product and type of retailing. In all cases differences between e-commerce and physical store are statistically significant (*p* < 0.005); Comparative between type of fish products within same group (E-commerce or Physical store); a, Significant differences (*p* < 0.005) compared to frozen fish; b, Significant differences (*p* < 0.005) compared to frozen seafood; c, Significant differences (*p* < 0.005) compared to cooked seafood; d, Significant differences (*p* < 0.005) compared to fish based ready-to-eat meals; e, Significant differences (*p* < 0.005) compared to fresh fish; f, Significant differences (*p* < 0.005) compared to fresh seafood; g, Significant differences (*p* < 0.005) compared to canned fish.

Regarding the type of retailing, “frozen fish” and “fresh fish” were also the categories with a higher relative increment in online purchases, followed by “frozen seafood” and “fresh seafood”. However, the category whose purchases increased the most in the physical store was “frozen fish” followed by “frozen seafood” and “fish-based ready-to-eat meals”, but with marked differences (Figure 5).

## 4. Discussion

The present study shows the impact of COVID-19-related lockdown on fishery purchases in a sample of more than five million people living in Spain. To date, no study has been published with such a large sample, not least with a pre- and post-pandemic comparison. 

The results show a significant increase in the purchase of all types of fishery products by Spanish households during the COVID-19 lockdown. These data are in concordance with the 2020 annual report carried out by the Government Household Food Consumption Panel [19]. According to this report, in the year 2020, there was a general increase in household food consumption, including fish products. This can be easily explained as many individuals that used to eat at least one meal away from home, such as many workers and their schoolchildren, found themselves having to do the shopping for all their daily meals. In addition, leisurely meals that were made in restaurants and bars, especially during the weekends, also changed to be made at home, with the exception of some restaurants that a few days after the declaration of the state of the alarm began to deliver meals [20]. Nevertheless, although an average rise of 2.3 kg of fish consumption in Spanish households was observed in the year 2020, as compared to 2019, according to the annual report carried out by the Government Household Food Consumption Panel, this increase was below the average of the rest of the food sector [19]. Moreover, figures of fish purchases in 2020 were still 6.9% lower than in 2013, in accordance with the downward trend in fish sales observed since 2008 in Spain. Nonetheless, the 45% increment in purchases of fish, seafood, and fish products observed in our study is much higher than the percentage destinated to this food group consumption outside the home in 2019 (14%), proving that intake of fishery products may have really increased during the lockdown and not just compensated for previous out-of-home consumption [3].

Newly released data for the 2021 annual report carried out by the Government Household Food Consumption Panel [21] indicate that household fish, seafood, and fish product consumption decreased by 8.5% compared to 2020. However, per capita consumption and percentage of total food purchases remained higher than in 2019 (an increase of 0.8% in per capita consumption and 1.2% in total food purchasing). This could indicate that the increment in fish and seafood purchases observed in the present study may be somewhat sustained over time. 

The generalized fear that supermarkets could run out of stock, especially during the first days of the lockdown, could explain why March and April were the months with the highest increase in fish, seafood, and fish products purchases. In fact, a publication by Gómez-Corona et al. described that food supplies (food stock and shortage products) were one of the major fears during the COVID-19 pandemic [22].

If we compare our data with the results found in other countries, it seems that the lockdown impact on fish purchases in Spain may have been different from other countries, as the majority of the studies carried out abroad reported a decrease in fish and seafood consumption [8].

When stratifying the sample by loyalty card holders’ categories, the finding of main interest is the significant difference between the retiree and the youngest populations in the relative increment of fish, seafood, and fish product purchases. These results are in accordance with another study carried out among Dutch adults, where researchers observed that the oldest age group was more likely to purchase more fish than usual during the lockdown as compared to younger populations [12].

Data from the ANIBES study on the consumption of the Spanish population before the pandemic already indicated that the younger population consumed a much lower amount of fish, seafood, and fish products than older adults (62.6 ± 70.4 vs. 81.1 ± 72.5 g/day) [7]. According to our results, this difference may have increased even more because of the pandemic, which may lead to a higher deviation from nutritional recommendations in the younger populations. In fact, although the purchases of fish, seafood, and fish products increased in all age groups during the lockdown, the long impact of this change may be different in the diverse age groups. In fact, according to the annual report carried out by the Government Household Food Consumption Panel, household fish consumption by the young population in 2021 is lower than in 2019, whilst it is higher in the retirees and older adults [3,21].

Data from the present study, however, are not sufficient to elucidate the reason why the youngest populations purchase less fish, seafood, and fish products than the retiree. Youngsters may have found difficulties in the preparation of some dishes at home if they were not used to preparing and cooking them. However, others might have seen an opportunity for learning or preparing more sophisticated recipes, as described by Romero-Arroyo et al. [23]. Besides, we also hypothesize that the retiree is the age group most concerned about cardiovascular diseases and perceive fish and derived products as a key group for prevention. In this regard, the American Heart Association recommends 1–2 seafood meals per week to reduce the risk of congestive heart failure, coronary heart disease, ischemic stroke, and sudden cardiac death, especially when seafood replaces the intake of less healthy foods [24]. Moreover, the possible relationship between vitamin D and the severity of the SARS-CoV-2 infection, especially among the most vulnerable population, may have also influenced the increase in fish and fish products consumption by a certain group of retirees, as an alternative to vitamin D synthesis from sun exposure [25]. However, another study carried out on elderly Japanese observed that there were more older adults that decreased their intake of fish than those who increased its consumption during the pandemic [26]. This reinforces the idea that the pandemic may have had different effects on different countries.

The large increase in e-commerce shopping among the retired group can be understood as some supermarket chains, such as the one surveyed in the present study, gave priority and helped their senior customers to do their shopping online. Nevertheless, it is still surprising that the group of people in which the digital divide is especially important [27] were the ones that most increased online shopping. This suggests that, with the will and necessary resources, this digital divide could be solved or at least reduced.

The fact that the increase in online shopping was higher than in-store purchases across all loyalty card holders’ categories, together with the recommendations of doing the shopping by just one member of the family and to do it once a week at most during the lockdown, may have also led to changes in shopping choices. In fact, many people make their decisions in real time at the supermarket, especially when it comes to fresh products, such as fish and shellfish. The shift from in-store to online shopping is especially noteworthy in a traditional Mediterranean country, such as Spain, and should be considered, as it may be a normalizing habit after the pandemic.

According to the type of products, it could be expected that “canned fish” would have the highest increase in sales because of its longer self-life and easiness of storing. However, “frozen fish” was the category with the highest relative increment of purchases, both in e-commerce and physical store retailing. These data are in accordance with the annual report carried out by the Government Household Food Consumption Panel report, where “frozen fish” was also the category whose purchases increased the most during the whole year 2020 [19]. Frozen fish is also considered non-perishable food and is organoleptically similar to fresh fish, although its storage requires space in the freezer. However, despite these numbers, according to the annual report carried out by the Government Household Food Consumption Panel, “fresh fish” was the type of fishery product with the largest purchases in 2020 and the second category whose sales increased the most [19], which is also in concordance with the present results. On the other hand, the type of product that increased its purchases the least was “ready-to-eat fish meals” which may be explained by the fact that people may have had more time for cooking at home [28]. These data should not go unnoticed as they may indicate that if some pandemic-related measures that provide more personal time for cooking persist, it could lead to healthier dietary habits, as most ready-to-eat meals are high in salt, sugar, and unhealthy fats.

In addition to all the above, another aspect that may have influenced the type of product purchased is the fact that not all food and food products were easily available at the supermarkets during the lockdown. It was revealed that during the pandemic, there was a significant decline in catches, imports, and exports of fresh seafood [29]. According to a study carried out in Spain, 12% of the participants reported having difficulties in finding fish [30].

### 4.1. Strengths of the Study

One of the main strengths of the study is the sample size. To our knowledge, the impact of COVID-19-related lockdown on so many people living in Spain has not been studied. Moreover, the diversity of the sample including youths, adults, families with babies, families with older children and retirees, is also noteworthy. Besides, the data come from more than 1500 establishments throughout the whole territory of Spain. Finally, the method of data collection, which is considered totally objective as it does not depend on the perception of the subject, is also a strength to highlight.

### 4.2. Weaknesses of the Study

The main limitation of the present study may be that, although loyalty card data capture what is bought, they cannot directly reveal exactly what is consumed by whom, nor can food waste be known [14]. Moreover, as age was directly related to the severity course of the COVID-19 illness [31], many relatives and volunteers did the groceries for the elderly so that they did not have to leave their homes and be exposed to the virus. This may have distorted some of the data by assigning purchases destinated to the “retired” group to other groups, especially in the case of physical store acquisitions.

## 5. Conclusions

There was a 45% general increase in purchases of fish, seafood, and fish products by Spanish households during the months of lockdown in Spain. Especially noteworthy is the significant difference between the retiree and the youngest populations in the relative increment of fish, seafood, and fish products purchases, much higher in the elderly. The use of e-commerce is also remarkable among the group of older age. These data are of special interest since events like the COVID-19 confinement can have a permanent impact on people’s dietary behavior, which should be monitored in the future.

## Figures and Tables

**Table 1:** Classification of loyalty card holders.

Category	Description	N		%	
		2019	2020	2019	2020
Retired	Age ≥65 years	919,223	805,701	16.7	15.7
Adults	Age 45–<65 years and no children in the household	835,256	809,928	15.2	15.7
Youth	Age 18–<45 years and no children in the household	924,412	913,044	16.8	17.7
Families with babies	Youngest child <6 years	586,088	572,224	10.6	11.1
Other families	Youngest child 6–30 years	1,643,137	1,495,821	29.8	29.1
Unclassified		603,169	549,098	10.9	10.7
TOTAL		5,511,285	5,145,816	100	100

## Data Availability

The data that support the findings of this study are available on request from the corresponding author. The data are not publicly available due to privacy or ethical restrictions.
